# Responses of *Japonica* Rice Quality Indicators and Starch Properties to Low Temperature at Different Periods of the Grain-Filling Stage in Cold Regions

**DOI:** 10.3390/foods15081355

**Published:** 2026-04-13

**Authors:** Mingyu Fan, Miao Hou, Fanxu Meng, Wenxuan Dai, Chuanming Yang, Hongyu Li

**Affiliations:** College of Agronomy, Heilongjiang Bayi Agricultural University, Daqing 163000, China; 15840511707@163.com (M.F.); 19586163323@163.com (M.H.); 17604669907@163.com (F.M.); 15845940640@163.com (W.D.); mosangbikezaizheli@haas.cn (C.Y.)

**Keywords:** *Japonica* rice, quality, starch, low temperature, grain-filling stage

## Abstract

Low temperature during grain filling is a major constraint affecting rice quality in cold regions. This study investigated how low temperature influences rice quality and starch characteristics at different periods of the grain-filling stage using two *Japonica* rice cultivars, Kenjing 7 (KJ7, moderate stress tolerance) and Kenjing 8 (KJ8, strong stress tolerance). Low-temperature treatments (17/13 °C, day/night) were applied during the early (5–11 days after anthesis), middle (12–18 days), and late (19–25 days) grain-filling stages and milling, appearance, nutritional, eating and cooking qualities as well as starch physicochemical properties were evaluated. Responses differed markedly between cultivars and treatment periods. Under low-temperature conditions, brown rice and milled rice rates of KJ8 increased during the early and middle grain-filling stages, whereas those of KJ7 declined during the late stage. Low-temperature stress increased protein, total starch, and amylose contents, while reducing gel consistency and the taste value of KJ7. Grain chalkiness increased significantly during the late stage, whereas during the early and middle stages, grain chalkiness, peak viscosity, and breakdown decreased and setback increased. Low temperature increased starch granule size and the proportions of short and intermediate chains of amylopectin, reduced medium-long and long chain and relative crystallinity, without altering starch crystalline type, and produced uneven starch particle surfaces with small pores. These effects were most pronounced during the late grain-filling stage. Overall, low temperature altered starch content and structure, thereby modifying pasting properties and ultimately leading to differences in rice quality.

## 1. Introduction

Rice (*Oryza sativa* L.) is one of the most important food crops worldwide, and nearly half of the global population depends on rice as a primary source of dietary energy. Food security therefore requires not only sufficient production but also stability and high quality. With improvements in living standards, rice production has gradually shifted from an exclusive emphasis on yield to a dual focus on yield and quality, making the stability of rice quality an increasingly important objective of agricultural development. Heilongjiang Province is the largest production region of high-quality *Japonica* rice in China. According to data released by the Heilongjiang Provincial Department of Agriculture and Rural Affairs, the planting area of *Japonica* rice in Heilongjiang Province exceeded 50 million mu in 2025, playing a critical role in ensuring national food security. However, Heilongjiang Province is located in a cold rice-growing region, where low-temperature damage is widespread, frequent, and severe. Although global warming has resulted in an overall increase in accumulated temperature across rice-growing regions, most areas of Heilongjiang Province are still expected to face substantial risks of cold damage before the end of the 21st century [1].

Rice is a tropical and subtropical crop, and the optimal temperature for most rice-growing regions ranges from 22 °C to 28 °C [2]. The grain-filling stage represents a critical period for the formation of rice yield and quality, during which temperature exerts a strong and direct influence on quality traits [3]. Under low-temperature conditions at this stage, net photosynthetic rate, maximum photochemical efficiency, and the activities of glutamic-pyruvic transaminase, glutamic-oxaloacetic transaminase, and starch branching enzyme (SBE) in rice leaves are reduced, thereby disrupting carbon and nitrogen metabolism and ultimately suppressing photosynthesis [4]. Low temperature during the grain-filling stage also delays the onset of grain filling and prolongs its duration, resulting in reduced grain plumpness and consequent declines in yield and quality [5,6]. Previous studies have extensively examined the effects of low temperature during the grain-filling stage on rice quality using cold-water irrigation, artificial climate chambers, and staggered sowing methods [6,7,8,9,10]. Low-temperature stress during grain-filling has consistently been shown to reduce rice eating and cooking quality [5,9,11], with particularly pronounced effects on appearance quality [11,12]. Low temperature induces abnormal starch granule development and poor filling of endosperm cells and tissues [13], thereby increasing grain chalkiness [5,11,14]. Segmented low-temperature treatments have further demonstrated that rice milling quality improves under low-temperature stress at one week of post-heading, chalkiness is reduced at two weeks of post-heading, and milling quality declines while chalkiness increases at the third week of post-heading [2]. Other studies have reported that, under low-temperature conditions, protein content increases during the grain-filling stage [5,8], decreases during the middle and late stages, and varies among cultivars during the early stage [15]. Dynamic low-temperature treatment of soft rice during grain filling has also shown that low temperature has no significant effect on protein content [9]. In contrast, Guo et al. [6] reported that low temperature reduced grain protein content but exerted no significant effects on other quality traits during the late grain-filling stage. Collectively, these studies indicate that the effects of low temperature during the grain-filling stage on rice quality depend on both the timing of stress exposure and variety [2,6].

The ratio of amylose to amylopectin and the molecular structure of starch are strongly influenced by temperature and light during the grain-filling stage [16]. Most studies have reported that low temperature during the grain-filling stage increases amylose content, while decreasing total starch and amylopectin contents [7,8]. However, other studies have suggested that the effect of low temperature on amylose content depends on the intrinsic amylose content of the cultivar [17]. During the grain-filling stage, low temperature increases setback viscosity and decreases peak, trough, final, and breakdown viscosities [18], as well as relative crystallinity [8,9,19]. Lower breakdown viscosity and higher setback viscosity are associated with reduced crystallinity, a decreased amylose-to-amylopectin ratio, lower gelatinization enthalpy, and increased starch granule size [9,19]. Under low-temperature conditions during the grain-filling stage, the proportions of short chains (DP 6–12) and intermediate chains (DP 13–24) increase, whereas the proportions of medium-long chains (DP 25–36) and long chains (DP > 37) decrease [10,17]. Reductions in medium-long amylopectin chains, crystallinity, and the amylose-to-amylopectin ratio, together with an increase in large starch granules, deteriorate the thermal and gelatinization properties of starch, resulting in decreased stickiness and elasticity of cooked rice [19]. However, the effects of low temperature on amylopectin chain-length distribution and RVA characteristics vary among cultivars during the grain-filling stage [2,10,17].

In the future, increasing demand for rice consumption, together with the rising frequency of extreme low-temperature events, will pose major challenges to rice production [20]. Although substantial progress has been made in elucidating the effects of low temperature on rice quality during the grain-filling stage, clear differences persist among cultivars in their responses to stress timing and intensity, and the mechanisms underlying starch responses to low temperature remain incompletely understood. Moreover, studies focusing on rice production systems in cold regions are still relatively limited. Therefore, two high-quality *Japonica* rice cultivars with contrasting stress tolerance, Kenjing 7 (KJ7) and Kenjing 8 (KJ8), developed by our research team, were selected as experimental materials. Based on the occurrence patterns and characteristics of low-temperature damage in Heilongjiang Province, we analyzed the effects of low temperature on rice quality and starch characteristics at different grain-filling stages, thereby providing a scientific basis for breeding cold-tolerant, high-quality rice varieties and grain quality improvement in cold regions.

## 2. Materials and Methods

### 2.1. Experimental Design

**Figure 1:**
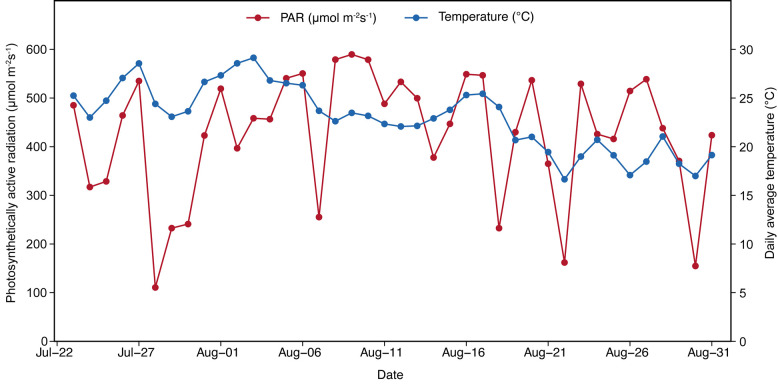
Daily average temperature and photosynthetically active radiation recorded between July and August 2022.

The experiment was conducted from 2022 to 2023 at the rice pot-culture base and in the artificial climate chamber of Heilongjiang Bayi Agricultural University. A two-factor completely randomized design was used. Factor A included two high-quality *Japonica* rice cultivars, KJ7 (moderate stress tolerance) and KJ8 (strong stress tolerance). Factor B comprised low-temperature treatments with four levels, as shown in Table 1. Pot cultivation was adopted throughout the experiment. Rice seeds were sown in mid-April, and seedlings were transplanted in mid-May into pots measuring 32 cm in height, 30 cm in top diameter, and 27 cm in bottom diameter. Three replicates were established, with 60 pots per replicate, four hills per pot, and three seedlings per hill. After flowering, plants of each cultivar were transferred from outdoor conditions to the artificial climate chamber to impose low-temperature stress according to the treatment schedule presented in Table 1. An intelligent temperature control system regulated chamber temperatures at 07:00 and 19:00 each day to generate day–night temperature differences. Temperature and humidity inside the climate chamber were continuously monitored using a temperature and humidity meter. Water and fertilizer management were maintained consistently between control and low-temperature treatments. A microclimate automatic observation system (RR-9100, Rainroot Scientific, Beijing, China) was used to record daily mean temperature and photosynthetically active radiation at the pot-culture base from July to August 2022 (Figure 1).

### 2.2. Sampling and Measurement

#### 2.2.1. Milling and Appearance Quality

After harvest, rice samples were stored for 3 months and then threshed using a small threshing machine. A brown rice machine (FC2K, SATAKE, Tokyo, Japan) and a milled rice machine (VP-32, YAMAMOTO, Yamagata, Japan) were used to process the samples into milled rice. Chalkiness rate, chalkiness degree, and broken rice rate were determined using an appearance quality identification analyzer (EM-1000, SATAKE, Japan), after which the rates of brown rice, milled rice, and head rice were calculated. All determinations were performed according to the National Standard of the People’s Republic of China (No. GB/T 17891-2017 [21]).

#### 2.2.2. Protein Composition

A precisely weighed 2.000 g sample of milled rice flour was used to determine nitrogen content using an automatic Kjeldahl nitrogen analyzer (K1100, Hanon, Jinan, China), and rice protein content was calculated using a conversion factor of 5.95. Protein components were determined using a sequential extraction method. Albumin, globulin, prolamin, and glutelin were extracted sequentially with distilled water, 5% sodium chloride solution, 70% ethanol solution, and 0.2% sodium hydroxide solution, respectively. The contents of the protein components were quantified using a modified Bradford assay kit. Specific procedures used here otherwise followed those described by Fan et al. [22].

#### 2.2.3. Eating and Cooking Quality

The eating and cooking quality of the rice was assessed using a rice taste analyzer (STA-1A, SATAKE, Japan). It is a near-infrared one, which accurately detects quantities of primary rice components. The computer then analyzes the data to display a cooked rice taste value between 0 and 100. The 30.0 g milled rice was put into an aluminum cup and washed by flowing water for 3 min. Water was added until the total weight of the milled rice and water was 72 g. The milled rice was soaked for 30 min at room temperature, and the cup was put into a rice cooker. Then, the sample was steamed for about 30 min and kept warm for 10 min. The cooked rice was mixed with a plastic scoop before it was cooled for 20 min. Two hours later, the eating and cooking rice was determined by the taste analyzer. Evaluation indices included aroma, gloss, integrity, taste, palatability, and comprehensive score.

#### 2.2.4. Starch Content and Gel Consistency

Amylose content, total starch content, and gel consistency were determined according to the National Standards of the People’s Republic of China: GB/T 15683-2008, GB 5009.9-2016, and GB/T 22294-2008 [23,24,25], respectively. Amylose content was determined by iodine colorimetric method. The rice flour was defatted by methanol, gelatinized by NaOH and colored with iodine solution. The absorbance was measured at 720 nm wavelength, and the amylose content was calculated according to the standard curve. The total starch content was determined by enzymatic hydrolysis. After degreasing with petroleum ether and removing soluble sugar with ethanol, the milled rice flour was hydrolyzed by Takadiastase at 55–60 °C and refluxed with hydrochloric acid in a boiling water bath. The declining sugar was determined by alkaline copper tartrate titration, and the total starch content was calculated by multiplying by 0.9. The 100 mg rice flour was put into the test tube, and thymol blue ethanol solution (0.2 mL, 0.025%) and KOH (2.0 mL, 0.2 mol/L) were added successively. After shaking well, the mixture was immediately heated in a boiling water bath for 8 min. After the end, it was taken out and cooled at room temperature for 5 min, followed by immersion in an ice water bath for 20 min. After cooling, it was placed in an incubator (25 ± 2 °C) for 1 h and then measured systematically. Amylopectin content was calculated as the difference between total starch content and amylose content.

#### 2.2.5. Pasting Properties of Rice Starch

We measured the pasting properties of rice starch using a rapid visco analyzer (RVA-4, Newport Scientific, Warriewood, Australia). Recorded parameters included peak viscosity, hot paste viscosity, cool paste viscosity, pasting temperature, breakdown viscosity, setback viscosity, peak time, and recovery viscosity. Specific procedures used here otherwise followed those described by Fan et al. [22].

#### 2.2.6. Structural Analysis of Amylopectin

The fine structure of amylopectin was determined by following the previous method with certain modifications [26]. Approximately 200 mg rice flour was incubated overnight at 37 °C in tricine buffer which contained protease (10 units/mL). The mixture was then centrifuged at 4000× *g* for 10 min to remove the supernatant and top light-yellow layer. The white layer at the bottom was the separated starch, which was washed three times with anhydrous ethanol and deionized water respectively. The 10 mg purified starch was dispersed in deionized water and reacted at 80 °C for 30 min. After cooling to 37 °C, the rice starch was debranched using isoamylase in sodium acetate buffer for 3 h. Subsequently, it was immersed in a water bath at 80 °C for 1 h. The debranched starch was collected after freeze-drying overnight. The 0.5 mg debranched starch was labeled with 8-amino-1,3,6-pyrenetrisulfonic acid and analyzed by using an MDQ Plus FACE System (AB SCIEX, Framingham, MA, USA), coupled with a solid-state, laser-induced fluorescence detector and an argon-ion laser as the excitation source.

According to the classification method of Hanashiro et al. [27], amylopectin chains were divided into four types: Fa (DP 6–12), Fb1 (DP 13–24), Fb2 (DP 25–36), and Fb3 (DP 37–100).

#### 2.2.7. Starch Particle Size Distribution

Starch granule size distribution was measured using a laser granulometer (Mastersizer 2000, Malvern, UK). Based on the principle of laser diffraction, the instrument measures particle sizes within a range of 0.1–2000 μm. Particle size distribution data were quantitatively processed using Mastersizer 2000 analysis software.

#### 2.2.8. Starch X-Ray Diffraction (XRD)

XRD analysis of starch was performed using an X-ray diffractometer (D8 Advance, Bruker, Germany) operated at 200 mA and 40 kV, with a diffraction angle (2θ) ranging from 4° to 40° (2θ) and a scanning speed of 0.02°. Relative crystallinity was calculated as the ratio of crystalline area to total area using MDI Jade 6 software.

#### 2.2.9. Morphology of Starch Granules

Starch samples were dried at 40 °C for 12 h and then evenly mounted on the sample stage. A gold film was applied by ion sputtering, after which starch granules were observed using a scanning electron microscope (S-4800, Fujifilm, Tokyo, Japan) at a magnification of 3000×.

### 2.3. Data Processing and Statistical Analysis

Excel 2016 was used to organize the experimental data, DPS 7.05 was applied for statistical analysis, and R-4.5.2 was used for plotting. Data in each graph are presented as mean ± standard error. The trends observed in 2022 and 2023 were consistent; therefore, this study primarily reports and analyzes the data from 2022.

## 3. Results

### 3.1. Rice Quality Analysis

#### 3.1.1. Milling Quality

**Figure 2:**
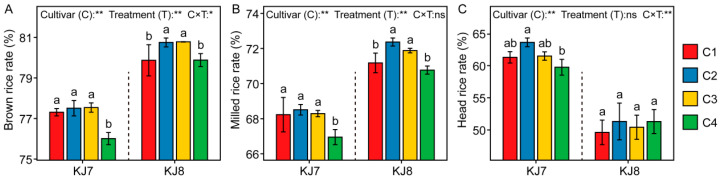
Milling quality in response to different treatments. (**A**) Brown rice rate; (**B**) Milled rice rate; (**C**) Head rice rate. Different lowercase letters indicate significant differences among treatments at the 5% level. *, **, and ns indicate significance at the 5% level, 1% level, and non-significance, respectively.

Cultivar, low-temperature treatment, and their interaction had highly significant effects on milling quality (Figure 2). In KJ7, the C2 and C3 treatments did not significantly affect milling quality, whereas the C4 treatment caused a significant reduction of 1.68% in brown rice rate and 1.88% in milled rice rate relative to the control. By contrast, in KJ8, the C2 and C3 treatments significantly increased both brown rice and milled rice rates. The head rice rate was comparatively less affected by low-temperature treatments across cultivars. These results indicate that responses of milling quality to low temperature varied between cultivars and depended on the timing of low-temperature exposure during the grain-filling stage.

#### 3.1.2. Appearance Quality

**Figure 3:**
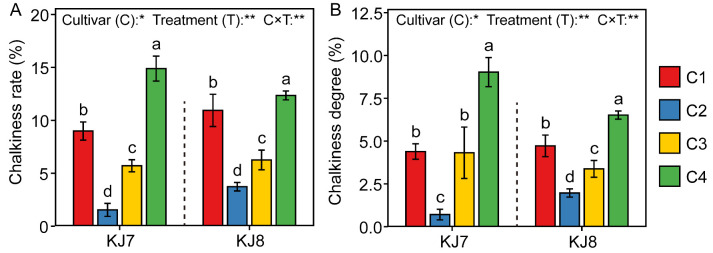
Appearance quality in response to different treatments. (**A**) Chalkiness rate; (**B**) Chalkiness degree. Different lowercase letters indicate significant differences among treatments at the 5% level. *, ** indicate significance at the 5% level, 1% level, respectively.

Cultivar, low-temperature treatment, and their interaction were found to have significant effects on appearance quality (Figure 3). For both cultivars, chalkiness rate and chalkiness degree followed the order C4 > C1 > C3 > C2. Under the C4 treatment, the chalkiness rate and degree of KJ7 increased significantly by 65.63% and 105.22% relative to the control, respectively, whereas those of KJ8 increased by 12.89% and 37.97%, respectively. By contrast, under the C2 treatment, the chalkiness rate and degree of KJ7 decreased significantly by 82.87% and 83.45%, respectively, and those of KJ8 decreased by 65.90% and 58.02%, respectively. These results indicate that, under low-temperature stress, rice appearance quality was markedly reduced during the late grain-filling stage, but substantially improved during the early and middle grain-filling stages. Overall, the appearance quality of KJ7 was more sensitive to low-temperature stress than that of KJ8.

#### 3.1.3. Protein Composition

**Figure 4:**
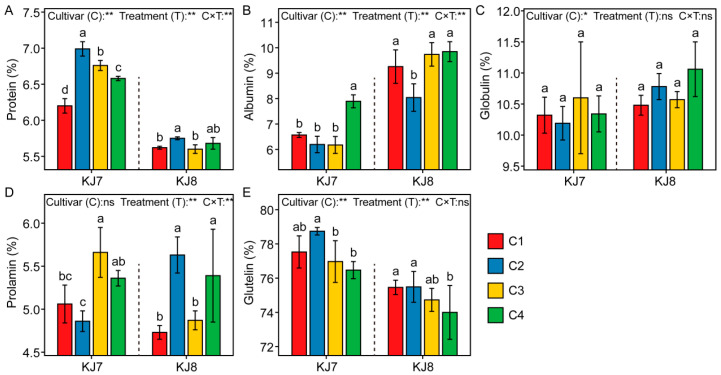
Protein composition in response to different treatments. (**A**) Protein content; (**B**) Albumin content; (**C**) Globulin content; (**D**) prolamin content; (**E**) Glutelin content. Protein component content is expressed as the percentage of each protein component relative to the total protein content. Different lowercase letters indicate significant differences among treatments at the 5% level. *, **, and ns indicate significance at the 5% level, 1% level, and non-significance, respectively.

Cultivar, low temperature, and their interaction significantly affected total protein and albumin contents (Figure 4). Significant differences in the content of total protein and protein components was observed between the two cultivars, with KJ7 exhibiting a significantly higher total protein content than KJ8. Relative to the control, under the C2 treatment, total protein content increased by 12.74% in KJ7 and by 2.31% in KJ8, whereas albumin content in KJ8 decreased by 13.30% and prolamin content increased by 19.03%. Under the C4 treatment, total protein and albumin contents of KJ7 increased by 6.13% and 20.52%, respectively, whereas in KJ8, prolamin content increased by 13.95% and glutelin content decreased by 1.93%. Overall, total protein content increased under low-temperature conditions during the grain-filling stage, with the C2 treatment producing the most pronounced effect. The responses of individual protein components to low temperature differed between the two cultivars.

#### 3.1.4. Eating and Cooking Quality

**Figure 5:**
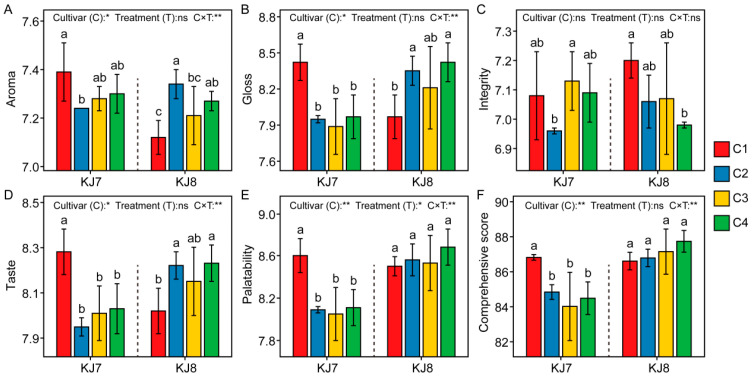
Eating and cooking quality in response to different treatments. (**A**) Aroma; (**B**) Gloss; (**C**) Integrity; (**D**) Taste; (**E**) Palatability; (**F**) Comprehensive score. Different lowercase letters indicate significant differences among treatments at the 5% level. *, **, and ns indicate significance at the 5% level, 1% level, and non-significance, respectively.

Cultivar and the interaction between cultivar and low temperature had significant effects on eating and cooking quality (Figure 5). Under low-temperature treatment, the eating and cooking quality of KJ7 exhibited a clear declining trend, with the comprehensive score decreasing by 2.28–3.23% relative to the control. By contrast, the aroma, gloss, and taste value of KJ8 showed increasing trends, and the comprehensive score of KJ8 was comparatively less affected by low temperature. These results indicate that the eating and cooking quality of KJ8 was more stable than that of KJ7 under low-temperature stress.

### 3.2. Analysis of Starch Characteristics

#### 3.2.1. Starch Content and Gel Consistency

**Figure 6:**
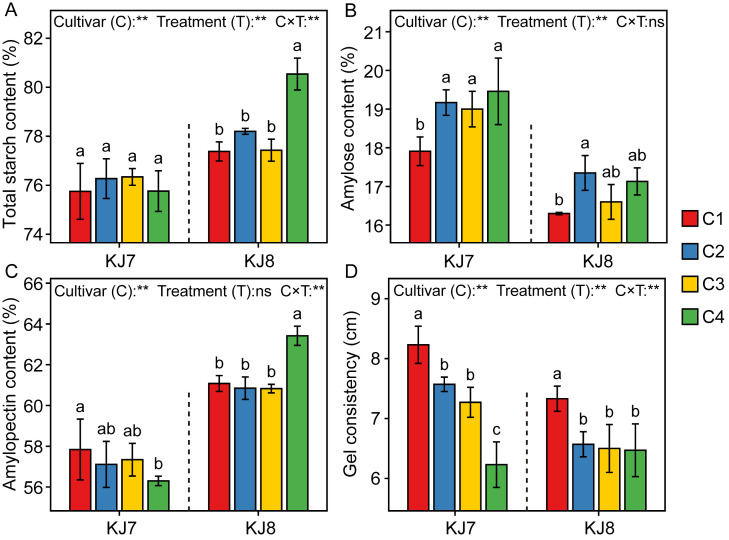
Starch content and gel consistency in response to different treatments. (**A**) Total starch content; (**B**) Amylose content; (**C**) Amylopectin content; (**D**) Gel consistency. Different lowercase letters indicate significant differences among treatments at the 5% level. **, and ns indicate significance at the 1% level, and non-significance, respectively.

Amylose content and gel consistency of KJ7 were significantly higher than those of KJ8, whereas total starch and amylopectin contents of KJ7 were significantly lower than those of KJ8 (Figure 6). Under low-temperature conditions, total starch and amylose contents generally increased in both cultivars. Relative to the control, the C2 treatment significantly increased amylose content in KJ7 and KJ8 by 7.04% and 6.44%, respectively. Under the C4 treatment, amylopectin content was significantly reduced by 2.66% in KJ7 but increased by 3.83% in KJ8. With delayed timing of low-temperature treatment, gel consistency exhibited a significant decreasing trend, with reductions of 8.02–24.30% in KJ7 and 10.37–11.73% in KJ8 (Figure 6D). These results indicate that low temperature during the late grain-filling stage exerted stronger effects on starch content and gel consistency than treatments applied at other stages.

#### 3.2.2. Pasting Properties of Rice Starch

**Figure 7:**
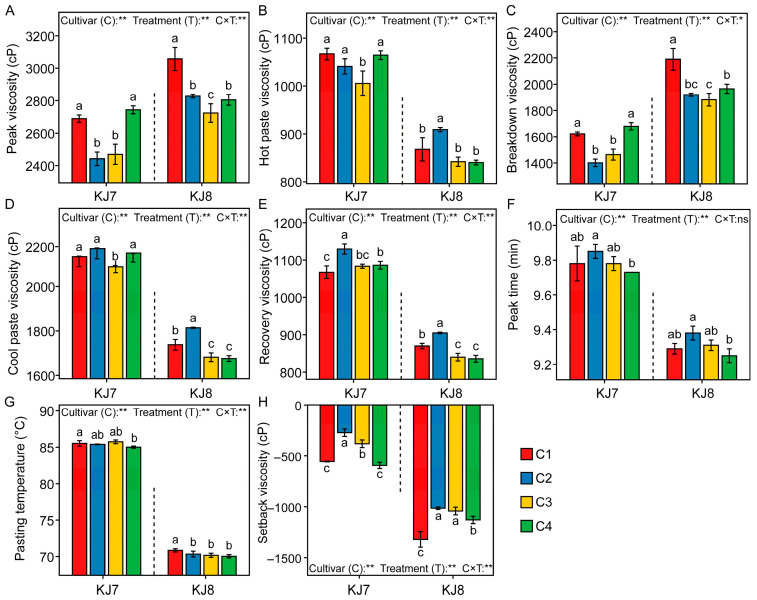
Analysis of pasting characteristics in response to different treatments. (**A**) Peak viscosity; (**B**) Hot paste viscosity; (**C**) Breakdown viscosity; (**D**) Cool paste viscosity; (**E**) Recovery viscosity; (**F**) Peak time; (**G**) Pasting temperature; (**H**) Setback viscosity. Different lowercase letters indicate significant differences among treatments at the 5% level. *, **, and ns indicate significance at the 5% level, 1% level, and non-significance, respectively.

Cultivar, low temperature, and their interaction significantly affected pasting parameters (Figure 7). The C2 and C3 treatments markedly reduced peak viscosity, pasting temperature and breakdown viscosity while increasing setback viscosity. Relative to the control, the C2 treatment significantly increased recovery viscosity in KJ7 and KJ8 by 5.81% and 4.02%, respectively. The C3 treatment significantly decreased the hot paste viscosity and cool paste viscosity of KJ7 by 5.75% and 2.11%, whereas the cool paste viscosity, recovery viscosity, and pasting temperature of KJ8 decreased by 3.20%, 3.41%, and 0.96%, respectively. The C4 treatment significantly increased the recovery viscosity of KJ7 and setback viscosity of KJ8, while significantly decreasing peak viscosity, breakdown viscosity, cool paste viscosity, recovery viscosity, and pasting temperature in KJ8. These results indicate that low-temperature treatments applied at different grain-filling stages altered starch pasting properties, with more pronounced effects observed in KJ8 than in KJ7.

#### 3.2.3. Fine Structure of Amylopectin

**Figure 8:**
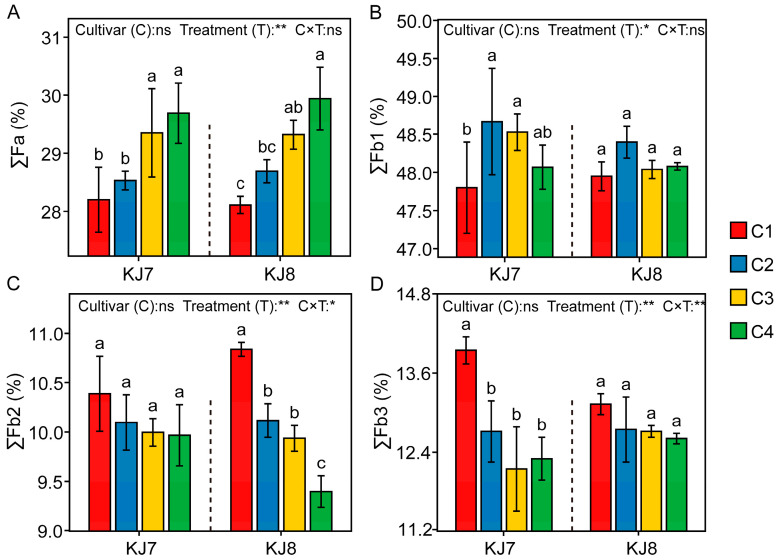
Analysis of chain length distribution of amylopectin in response to different treatments. (**A**) ∑Fa; (**B**) ∑Fb1; (**C**) ∑Fb2; (**D**) ∑Fb3. ∑Fa, ∑Fb1, ∑Fb2, and ∑Fb3 represent the relative percentages of Fa (DP 6–12), Fb1 (DP 13–24), Fb2 (DP 25–36), and Fb3 (DP 37–100), respectively. Different lowercase letters indicate significant differences among treatments at the 5% level. *, **, and ns indicate significance at the 5% level, 1% level, and non-significance, respectively.

The differences in chain length distribution of amylopectin between the two cultivars were relatively small, whereas low-temperature treatment exerted significant effects on chain length distribution (Figure 8). Overall, under low-temperature stress, the proportions of Fa and Fb1 increased, while the proportions of Fb2 and Fb3 decreased. Relative to the control, under the C4 treatment, the Fa proportion of KJ7 increased significantly by 5.28% and the Fb3 proportion decreased significantly by 11.97%, whereas in KJ8, the Fa proportion increased significantly by 6.51% and the Fb2 proportion decreased significantly by 13.28%. These results demonstrate that low temperature had a greater effect on the proportion of Fb1 during the early grain-filling stage and exerted significant effects on the proportions of Fa, Fb2, and Fb3 during the late grain-filling stage.

**Figure 9:**
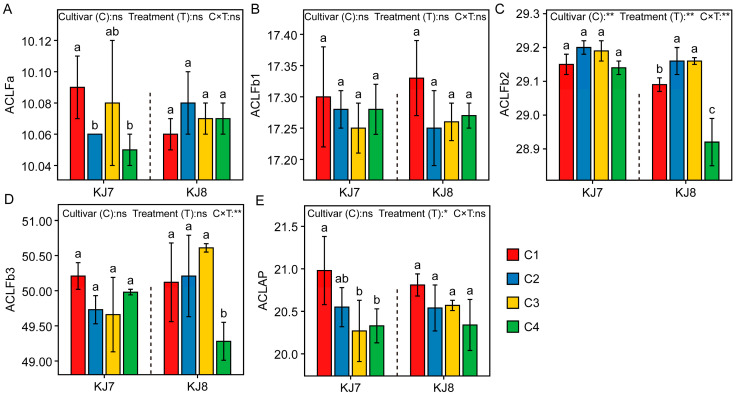
Average chain length of amylopectin in response to different treatments. (**A**) ACLFa; (**B**) ACLFb1; (**C**) ACLFb2; (**D**) ACLFb3; (**E**) ACLAP. ACLFa, ACLFb1, ACLFb2, ACLFb3, and ACLAP represent the average chain lengths of Fa, Fb1, Fb2, Fb3, and total amylopectin, respectively. Different lowercase letters indicate significant differences among treatments at the 5% level. *, **, and ns indicate significance at the 5% level, 1% level, and non-significance, respectively.

Low-temperature treatment had relatively small effects on the average chain length (ACL) of amylopectin (Figure 9). In KJ7, ACLFa was significantly reduced under the C2 and C4 treatments, and the ACL of total amylopectin was significantly decreased under the C3 and C4 treatments. In KJ8, ACLFb2 was significantly increased under the C2 and C3 treatments, whereas both ACLFb2 and ACLFb3 were significantly reduced under the C4 treatment. Overall, low temperature during the late grain-filling stage exerted a greater effect on the ACL of amylopectin than treatments applied at earlier stages.

#### 3.2.4. Size Distribution of Starch Granules

**Figure 10:**
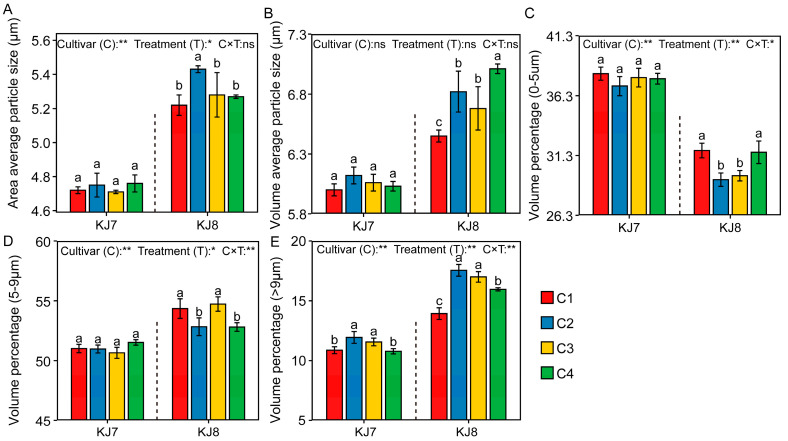
Size distribution of starch granules in response to different treatments. (**A**) Area average particle size; (**B**) Volume average particle size; (**C**) Volume percentage (0–5 μm); (**D**) Volume percentage (5–9 μm); (**E**) Volume percentage (>9 μm). Different lowercase letters indicate significant differences among treatments at the 5% level. *, **, and ns indicate significance at the 5% level, 1% level, and non-significance, respectively.

The starch particle size of KJ8 was significantly larger than that of KJ7 (Figure 10). Low-temperature treatment increased the area-average and volume-average starch particle sizes of KJ8, as well as the volume proportion of granules with d > 9 μm, while significantly reducing the volume proportion of granules with d < 9 μm. Under the C2 treatment, the area-average particle size, volume-average particle size, and volume proportion of granules with d > 9 μm in KJ8 increased significantly by 4.01%, 5.74%, and 25.99%, respectively, whereas the volume proportions of granules with sizes of 0–5 μm and 5–9 μm decreased significantly by 7.69% and 2.80%, respectively. By contrast, low-temperature treatment exerted relatively small effects on the starch granule size distribution of KJ7.

#### 3.2.5. Relative Crystallinity of Starch

**Figure 11:**
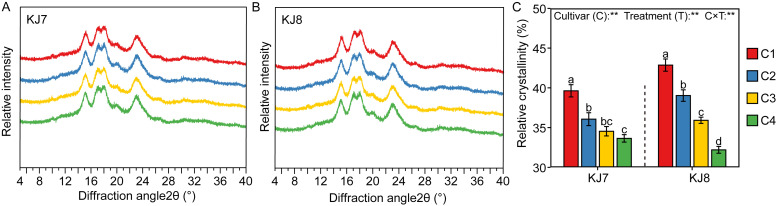
Analysis of XRD profiles and relative crystallinity of starch among different treatments. (**A**) Relative intensity of KJ7; (**B**) Relative intensity of KJ8; (**C**) Relative crystallinity. Different lowercase letters indicate significant differences among treatments at the 5% level. ** indicate significance at the 1% level.

Both cultivars exhibited strong diffraction peaks at 2θ angles of 15° and 23°, as well as continuous double peaks at 17° and 18° (Figure 11A,B). The XRD patterns of both cultivars under all treatments were of the A-type, indicating that low-temperature stress did not alter the crystalline type of rice starch. Relative crystallinity of the two cultivars ranged from 32.20% to 42.86%, with KJ8 exhibiting higher relative crystallinity than KJ7 (Figure 11C). Under low-temperature stress, the relative crystallinity of KJ7 and KJ8 decreased by 9.13–15.11% and 8.94–24.87%, respectively. The effect of low temperature on relative crystallinity was greatest during the late grain-filling stage.

#### 3.2.6. Starch Granule Surface Morphology

**Figure 12:**
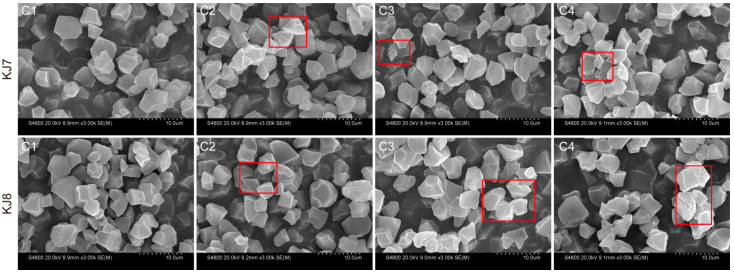
Starch granule morphology under different treatments. The starch granules within the red boxes disply the rough surface.

Starch granules from all treatments exhibited similar overall morphology and were predominantly irregular polyhedra (Figure 12). In the control, the surface of starch granules was generally smooth, whereas under low-temperature treatment, small pores appeared and surface roughness increased. Compared with the C2 treatment, starch granules subjected to the C3 and C4 treatments showed a greater degree of surface damage. Similar patterns of surface morphological changes were observed in both cultivars.

### 3.3. Correlation Analysis Between Rice Quality and Starch Characteristics

**Figure 13:**
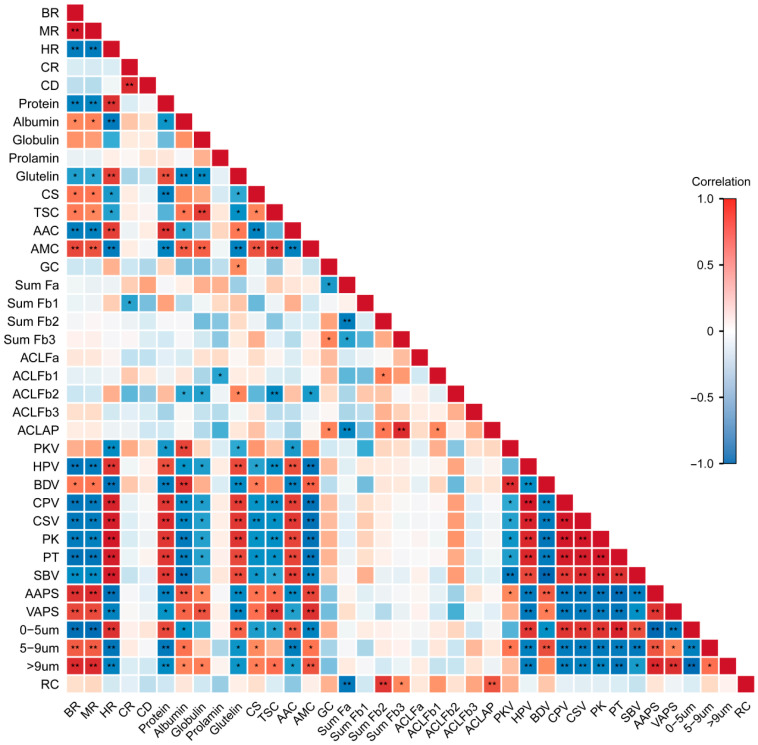
Heat map of correlations between rice quality and starch characteristics. BR, brown rice rate; MR, milled rice rate; HR, head rice rate; CR, chalkiness rate; CD, chalkiness degree; CS, comprehensive score; TSC, total starch content; AAC, amylose content; AMC, amylopectin content; GC, gel consistency; Sum Fa, Sum Fb1, Sum Fb2, Sum Fb3, proportion of Fa, Fb1, Fb2, Fb3 chain; ACLFa, ACLFb1, ACLFb2, ACLFb3, ACLAP, average chain lengths of Fa, Fb1, Fb2, Fb3, total amylopectin; PKV, peak viscosity; HPV, hot paste viscosity; BDV, breakdown viscosity; CPV, cool paste viscosity; CSV, recovery viscosity; PK, peak time; PT, pasting temperature; SBV, setback viscosity; AAPS, area-average particle size; VAPS, volume-average particle size; RC, relative crystallinity. *, ** indicate significance at the 5% level, 1% level, respectively.

Correlation analysis revealed that milling quality was significantly and highly significantly associated with protein content, comprehensive score, starch content, RVA pasting parameters, and starch granule size distribution (Figure 13). Protein content exhibited significant and highly significant correlations with comprehensive score, starch content, ACL of amylopectin, RVA pasting parameters, and starch granule size distribution. Similarly, the comprehensive score was significantly and highly significantly correlated with starch content, RVA pasting parameters, and starch granule size distribution. Starch content also showed significant and highly significant correlations with the ACL of amylopectin, RVA pasting parameters, and starch granule size distribution. Furthermore, amylopectin fine structure was significantly and highly significantly correlated with relative crystallinity, whereas RVA pasting parameters were significantly and highly significantly correlated with starch granule size distribution. Collectively, these results demonstrate that starch pasting properties are tightly linked to rice quality and starch structural characteristics.

## 4. Discussion

### 4.1. Effect of Low Temperature on Rice Quality

Northeast China is one of the major cold-region rice-growing areas in the country. Compared with other regions, rice production in Northeast China is more widely, frequently, and severely affected by cold damage [28]. Previous studies have demonstrated that the two weeks following heading constitute the most critical period for rice quality responses to extreme temperature stress. Milling quality has been shown to improve under low-temperature treatment during the first week post-heading but to decline during the third week post-heading [2]. Mild low-temperature stress during the grain-filling stage has also been reported to reduce the milling quality of hybrid rice restorer lines to varying extents [17]. In the present study, low-temperature treatment significantly increased brown rice and milled rice rates of KJ8 during the early and middle grain-filling stages, whereas these rates were significantly reduced in KJ7 during the late grain-filling stage. This indicates that milling quality responses to low temperature differed among cultivars and depended on the timing of exposure during the grain-filling stage [2]. Grain appearance quality is primarily determined by starch accumulation. During the grain-filling stage, low-temperature stress leads to loose packing of starch granules and the formation of larger intergranular air spaces, thereby promoting chalkiness formation [29]. Consistent with the findings [2], the present study showed that chalkiness was significantly reduced under low temperature during the early and middle grain-filling stages, whereas it was significantly increased during the late stage. Low-temperature stress after heading markedly reduced both maximum and average grain-filling rates and prolonged the overall grain-filling process [30]. During the early grain-filling stage, a relatively slow grain-filling rate, together with sufficient assimilate supply from leaves, allowed orderly translocation from source to sink, thereby producing well-filled grains with low chalkiness. Under low-temperature stress, however, leaf photosynthetic capacity declined [2], organic matter synthesis in rice plants was suppressed, and endosperm cell division and starch granule proliferation were inhibited, ultimately impairing grain filling and reducing appearance quality [9].

Extensive studies have investigated the effects of low temperature on rice protein content, but the results vary from expert to expert. Some studies concluded it increased protein levels [5,8,11,31], others showed its decreased tendency [3,6], and some even indicated that there is no significant effect [9]. In the present study, low-temperature stress generally enhanced protein content across different periods of the grain-filling stage. This may be because low temperature promotes the translocation of soluble protein from leaves to grains, thereby accelerating protein accumulation in grains. Further analysis suggests that the effect of low temperature on protein during the filling stage is related to the intrinsic stress tolerance and baseline protein levels, and the timing, magnitude and duration of low-temperature stress. For example, the impact of low temperature on protein content was smaller in the more stress-tolerant cultivar KJ8 than in KJ7. Some studies also found that grain protein concentration tends to decrease with increasing stress duration and decreasing temperature [28]. Analysis of protein components further indicated that responses of different protein components to low temperature differed among cultivars, which is consistent with findings reported by Wang et al. [32] from low-temperature experiments conducted during the booting stage. Among rice quality traits, eating and cooking quality is particularly important because it directly influences market price and consumer acceptance [33]. Previous studies involving different rice types and employing various stress methods have consistently demonstrated that low-temperature stress during the grain-filling stage reduces eating and cooking quality [5,9,11]. In the present study, the aroma, gloss, integrity, taste, palatability and comprehensive score measured by the instrument were used to comprehensively evaluate the eating and cooking quality of rice. The research showed that low-temperature treatment significantly reduced the eating and cooking quality of KJ7, whereas it had no significant effect on KJ8, indicating that KJ8 exhibited greater palatability stability under low-temperature stress.

### 4.2. Effects of Low Temperature on Starch Characteristics

During the grain-filling stage, the ratio of amylose to amylopectin and the molecular structure of starch are strongly influenced by temperature and light conditions [16]. Previous studies have demonstrated that low temperature increases amylose content during grain filling under stress conditions such as cold-water irrigation [7], artificial climate chambers [6,8,9], and field low-temperature exposure [10]. In the present study, amylose content increased under low temperature, which may be associated with enhanced activity of granule-bound starch synthase (GBSS) [34,35,36]. The total starch and amylopectin contents of KJ8 increased markedly, whereas the amylopectin content of KJ7 decreased under low temperature during the late grain-filling stage. Compared to amylose, amylopectin synthesis is a complex process regulated by multiple enzymes, including ADP-glucose pyrophosphorylase (AGPase), GBSS, soluble starch synthase (SSS), SBE, and debranching enzyme (DBE) [37]. Low temperature alters the activities of these enzymes, thereby affecting amylopectin accumulation [38]. Starch pasting properties are jointly influenced by amylose content and the side-chain distribution of amylopectin [39]. In the present study, peak viscosity, breakdown viscosity, and pasting temperature were markedly reduced, whereas setback viscosity was increased under low-temperature treatment during the early and middle grain-filling stages. Differences in chain length distribution of amylopectin lead to variation in gelatinization properties, pasting temperature, and relative crystallinity [16,40]. Consistent with previous research findings [10,17], low temperature during grain filling increases the proportion of short amylopectin chains and reduces the proportion of long chains that form stable double-helical structures (Figure 8). Because the temperature required for complete dissociation of short chains is lower than that required for long chains [3,41], low temperature consequently lowers the pasting temperature (Figure 7G).

In the present experiment, low-temperature treatment increased area-average and volume-average starch particle sizes and the proportion of large-sized granules (d > 9 μm). Low-temperature stress applied 3 days after flowering increased the proportion of large-sized granules (5–20 μm) in *Japonica* rice. It is possible that low-temperature stress after anthesis may suppress the division of large-sized granules, thereby increasing the amount of large-sized granules [8]. At the same time, the effects of low temperature on starch granule size distribution were significantly greater in KJ8 than in KJ7. Furthermore, low temperature affected milling quality, eating and cooking quality, protein content and amylose content differently between the two cultivars, indicating that cultivar-specific responses play an important role in determining the effects of low temperature on rice quality and starch properties during the grain-filling stage [2,8,10,17]. In the present study, starch from both cultivars exhibited an A-type crystalline pattern, and the relative crystallinity was significantly reduced under low-temperature conditions, which is consistent with previous reports [8,9,19]. Growth environment and management measures do not alter the crystalline type of starch [42,43,44]. However, environmental conditions can induce variation in starch granule morphology [42,45]. Starch granules typically exhibit irregular, angular shapes with smooth surfaces, whereas micropores appear on granule surfaces under low-temperature conditions [10]. The findings of the present study confirmed this conclusion. This phenomenon has been attributed to slowed amyloplast development under low temperature, which results in uneven granule surfaces [19,46]. The present study also found low temperature exerted stronger effects on starch content, gel consistency, amylopectin fine structure, relative crystallinity, and starch granule morphology during late periods than during other periods, indicating that low temperature during the late grain-filling stage (19–25 days after anthesis) had a greater impact on starch characteristics.

Under low-temperature stress, plants initially respond through transcriptional regulation by activating cold-responsive genes and synthesizing stress-related proteins, which subsequently influence the synthesis of related metabolites. Starch synthesis is jointly regulated by enzymes such as AGPase, GBSS, SSS, SBE, and DBE [37]. However, how the activities of these starch synthesis-related enzymes and the expression levels of their regulatory genes change under low-temperature stress during the grain-filling stage, and how these processes interact, remain unclear. In future studies, multi-omics approaches will be integrated to identify differential metabolites and gene expression patterns associated with starch synthesis, construct regulatory networks, and further elucidate the mechanisms underlying the effects of low temperature on starch formation in *Japonica* rice from phenotypic, physiological, and molecular perspectives in cold regions.

### 4.3. Correlation Between Rice Quality and Starch Characteristics

Starch accounts for approximately 95% of the dry weight of rice grains and plays a crucial role in determining eating and cooking quality [17,47]. Amylose content and the amylose-to-amylopectin ratio play important roles in determining eating and cooking quality [3,41]. In present research, significantly, the taste value was negatively correlated with amylose content and positively correlated with amylopectin content. Amylose chains can co-crystallize with amylopectin chains and are distributed within amylopectin microcrystals. During cooking, these amylose molecules enhance the thermal stability of starch crystallites, restrict starch swelling and leaching, and ultimately result in a harder texture and reduced stickiness of cooked rice [48]. RVA profiles, which simulate the cooking process of rice, directly reflect changes in starch gelatinization behavior and viscosity during heating and cooling and are recognized as key indicators for evaluating eating and cooking quality [49]. Correlation analysis showed that the RVA pasting parameters were strongly correlated with other quality traits. The taste value was significantly correlated with protein content, starch content, RVA pasting parameters, and starch granule size distribution. Therefore, reduced palatability under low-temperature stress may be attributed to increased amylose and protein contents, together with decreased pasting viscosity and gel consistency. The present study demonstrated a significant negative correlation between relative crystallinity and the proportion of short chains in amylopectin. Under low-temperature treatment, the increased proportion of short amylopectin chains disrupts the continuity of double-helical structures across crystalline regions and consequently reduces relative crystallinity [19]. Combined with previous studies [16,19,50] and correlation analysis (Figure 13), the present study suggests that low temperature during the grain-filling stage alters starch content and structure, leading to changes in pasting properties and ultimately resulting in differences in rice quality.

## 5. Conclusions

The responses of quality traits and starch characteristics to low-temperature stress at different grain-filling periods differed among cultivars. The effects of low temperature on rice quality were more pronounced in KJ7 than in KJ8. Among all quality traits, appearance quality was the most strongly affected by low temperature, with appearance quality improving during the early and middle grain-filling stages, but reducing during the late stage. In general, low-temperature treatment increased protein, total starch, and amylose contents in both cultivars, while significantly reducing gel consistency and decreasing the taste value of KJ7. Effects of low temperature on starch characteristics were stronger during the late grain-filling stage than during other stages. Low-temperature treatments applied at different grain-filling stages increased the starch granule size and the proportions of short and intermediate amylopectin chains, decreased relative crystallinity, and induced the formation of small pores on starch granule surfaces. Based on the results of the entire study, these findings indicate that low temperature during the grain-filling stage alters starch content and structure, thereby modifying pasting properties and ultimately leading to differences in rice quality.

## Figures and Tables

**Table 1:** Overview of experimental treatments.

Treatment	5–11 Days After Anthesis	12–18 Days After Anthesis	19–25 Days After Anthesis
C1 (CK)	Outdoor	Outdoor	Outdoor
C2	Climate chamber 17/13 °C (day/night)	Outdoor	Outdoor
C3	Outdoor	Climate chamber 17 °C/13 °C (day/night)	Outdoor
C4	Outdoor	Outdoor	Climate chamber 17 °C/13 °C (day/night)

CK: Control Check.

## Data Availability

The original contributions presented in this study are included in the article. Further inquiries can be directed to the corresponding author.
